# Accessibility to specialist palliative care services in Germany: a geographical network analysis

**DOI:** 10.1186/s12913-023-09751-7

**Published:** 2023-07-24

**Authors:** Daniela Gesell, Farina Hodiamont, Claudia Bausewein, Daniela Koller

**Affiliations:** 1 Department of Palliative Medicine, LMU University Hospital, LMU Munich, Munich, Germany; 2 Institute of Medical Data Processing, Biometrics and Epidemiology (IBE), Faculty of Medicine, LMU Munich, Munich, Germany

**Keywords:** Accessibility, Specialist palliative care, Geographical network analysis, GIS

## Abstract

**Background:**

The need for palliative care will increase over the next years because of the rise in deaths from chronic illness and demographic changes. The provision of specialist palliative care (SPC) in Germany (palliative care units (PCU), specialist palliative home care (SPHC) teams and palliative care advisory (PCA) teams) has been expanded in recent years. Despite the increasing availability, there is still insufficient coverage with long travel times. The aim was to describe the spatial distribution of SPC services in Germany, to calculate the potential accessibility of facilities and to assess potential spatial under-provision.

**Methods:**

Retrospective cross-sectional study with regional analysis of SPC services in Germany. Addresses of SPC services registered online were geocoded, accessibility and network analyses were conducted, and proportion of the population living up to 60 minutes driving time were calculated.

**Results:**

A total of 673 facilities were included. Their distribution is heterogeneous with every fourth of the 401 districts (110/401; 27.4%) lacking a SPC service. In half of the area of Germany the existing PCU and SPHC teams are within reach of 30 minutes, with nearly 90% of the population living there. Hospitals providing PCA teams can be reached within 30 minutes in 17% of the total area with provision for 43% of the population.

**Conclusions:**

A high coverage of SPHC teams and PCU indicates a good spatial distribution in Germany but no complete adequate provision of SPC services, especially for PCA teams. There is a persistent need for further implementation of hospital PCA teams.

**Supplementary Information:**

The online version contains supplementary material available at 10.1186/s12913-023-09751-7.

## Background

The World Health Organization defines palliative care as “a crucial part of integrated, people-centred health services. Relieving serious health-related suffering, be it physical, psychological, social, or spiritual, is a global ethical responsibility.”[1] The global health community has the responsibility and the opportunity to ensure that all people, irrespective of their financial situation, have access to palliative care and pain relief for life-threatening and life limiting health conditions and end-of-life care [2]. In Germany, every citizen is by law entitled to specialist palliative care (SPC) [3]. While generalist palliative care is part of general medical care, often offered by general practitioners, SPC has higher requirements in terms of qualifications and is delivered by a multiprofessional team [4]. These teams care for patients with complex symptom and problem burden, regardless of their diagnoses and the particular stage of a disease. They primarily focus on the needs of patients and their relatives. Comprehensive support in pain management and symptom control, as well as in ethical issues is intended to preserve patients’ autonomy and quality of life at the end of life [5–7].

In the context of the German health system, SPC is provided in three settings. Palliative care units (PCU) offer specialist inpatient care, usually as a ward within a hospital. Specialist palliative home care (SPHC) teams provide SPC to patients at home, they support their families and correspond to general practitioners and hospice services. Palliative care advisory (PCA) teams support healthcare professionals on hospital wards not specialised in palliative care. They are available to patients in all general wards of hospitals as needed in the treatment of their progressive, life-limiting disease. The teams are requested by the physicians or nursing staff of the corresponding wards and offer the possibility of a qualified palliative medical and nursing consultation. They are often the first entry point into SPC [4, 8]. Which type of SPC is provided should depend on the individual patients’ needs. While the different SPC services can work hand-in-hand, they are most often not interchangeable. In addition to SPC services, there are inpatient hospices and hospice services consisting of specially qualified nurses, supported by primary care physicians, volunteers and other professions [8, 9]. All of these settings constitute palliative care in Germany.

The availability and use of SPC has increased significantly in Germany in recent years but there is still no nationwide coverage [8, 10] and further expansion of palliative care services is needed in order to fill spots of potential under-provision [11]. Although the integration of SPC is already being realized in many regions, there is concern that some areas may be underserved especially in rural areas with no or very few services [10] and difficult access to existing services [12]. Understanding regional differences in the context of potential geographic access to facilities is critical to identify gaps or inequities in service coverage [13]. Previous studies have mainly focused on individual federal states or evaluated health insurance data for certain settings, such as home care teams [14, 15]. Wiese et al. examined the distribution of SPC in Germany in 2010, without considering population density, and concluded that there is an existing need for nationwide development of SPC [16]. There is no current study that considers the country as a whole and includes the population structure. To get an overview of the actual spatial coverage of SPC facilities in Germany for both urban and rural areas, it is necessary not only to describe the spatial distribution of services but also to focus on the potential geographical accessibility and regional distances. Therefore, the aim of this paper is to describe the spatial distribution of SPC services in Germany, to calculate the potential accessibility of facilities and to assess potential spatial under-provision in serving areas of SPC services.

## Methods

### Study design

We conducted a retrospective study with routinely-collected data following the RECORD (REporting of studies Conducted using Observational Routinely-collected health Data) statement [17].

### Setting

Germany has about 85 million inhabitants and is rather densely populated with approximately 240 inhabitants/km^2^. The spatial organization consists of 16 federal states, 401 districts and independent cities, 11,135 communities and 8,725 postal code areas (01/01/2022) [18]. For this study we used data on postal code and district level.

### Data sources/variables

Two sets of data were included in the analyses, providing information on the palliative services and on the regions in which they are based. The first data source comprises addresses of SPC services in Germany. Individual facilities were identified through the ‘Directory for Hospice and Palliative Care’ which is accessible online. The Directory is a web-based freely accessible data platform set up and run by the German Association for Palliative Medicine for voluntary registration of services and contains a large part of the palliative care services registered in Germany [19]. All PCU, SPHC teams, and PCA teams that care for adult patients and were registered in the Directory as of December 2020 were included. We included these three settings because comprehensive symptom control to patients is provided by multidisciplinary teams of specialist palliative care professionals.

For geographic information, we included freely accessible base maps for German districts from the Service Center of the Federal Agency for Cartography and Geodesy (Bundesamt für Karthographie und Geodäsie, BKG) [18] and data of the current spatial monitoring from the Federal Institute for Building, Urban Affairs and Spatial Research for the regional statistical calculations. The variables used comprise the number of inhabitants, population density, and settlement structures [20]. The maps are available as open data.

### Data preparation and categorization

The addresses of the SPC services were geocoded in the reference system UTM32/ETRS89 using the geocoder of the BKG to generate points of interest (POI). Subsequently, the data were aggregated at district level. To identify regional differences, first an urban-rural comparison was analysed with two types of urban and rural structures each: major cities and urban districts as well as rural districts with densification trends and low populated rural districts,[21, 22] hereafter called “urban” and “rural”, combining the two first and two last definitions. Population density was categorized according to the classification of the degree of urbanization of the Statistical Office of the European Union [23]. For descriptive analysis, the number of services per district was calculated per 100,000 inhabitants to achieve a reference, independent of district size.

### Statistical methods

Accessibility analysis was conducted based on the calculation of travel time with the geographic information system plugin Openrouteservice (ORS) Tools. ORS Tools provide access to open route service routing functionalities like isochrones and matrix calculations [24]. We used digital route data and geocoded POI.

There are no official distances or times defined in which SPC services need to be reachable. Therefore, we used travel times as identified in the literature and used car travel times with intervals of 15, 30 and up to a maximum of 60 minutes for the accessibility isochrones in accordance to other international studies [25–27]. As an empirically derived value, we defined 60 minutes as the threshold for accessibility for transport to the nearest palliative care service. The proportion of areas where a SPC service cannot potentially be reached within a maximum of 60 minutes was then calculated.

In addition to potential accessibility, we calculated average travel times that people would need from their homes to reach a SPC service or, for home care, the SPHC team has to travel to the patient. For this, we used the geographic centroids of all postal codes approximating as the home starting point and conducted a network analysis. The number of residents of each postal code area was included in the analysis. For each postal code, the nearest service was identified, the travel time by car was determined, and average travel times were calculated. Only the nearest service was considered for each postal code, regardless of administrative borders. The distances of postal codes centroids to services were calculated with ArcGIS Network Analyst in ArcGIS Pro. The network dataset used for this was collected from the ArcGIS Online Cloud service [28].

All cartography and analyses were conducted using QGIS (Vers. 3.10), ESRI ArcGIS Pro (Vers. 2.8) and IBM SPSS Statistics (Vers. 26).

## Results

After removing duplicates, a total of 673 German SPC services were included, comprising 317 PCU, 289 SPHC teams and 67 PCA teams in hospitals. There are 291 of 401 districts with at least one SPC service. In 167/291 districts (56.3%), the SPC services are in urban areas and in 124/291 districts (42.6%) in rural areas (see additional file 1). 110/401 districts (27.4%) have no SPC service.

Almost half a million people (for PCU), more than 10 Mio people (for PCA teams) and almost 250,000 people (for SPHC teams) live more than an hour away from their next service (see Table 1). These people are mainly located in western Schleswig-Holstein, eastern Mecklenburg-Western Pomerania, in the low populated districts of Prignitz and Uckermark in Brandenburg and in the moderately populated Hochsauerlandkreis in North Rhine-Westphalia as well as in parts of southwestern Baden-Württemberg. People living more than an hour to the next PCA team are located particularly in (north-) eastern regions in Saxony-Anhalt, Saxony, Thuringia, in west of Brandenburg, in west of Schleswig-Holstein, in east of Lower Saxony as well as in west of Hesse, in south of North Rhine-Westphalia, in east of Bavaria and in north and south of Baden-Württemberg.

**Table 1:** Percentage and total number of residents living in service areas of different driving times to palliative care units, palliative home care teams and palliative care advisory teams

Travel time (in minutes)	Palliative care units		Palliative care advisory teams		Specialist palliative home care teams	
	*n*	%	*n*	%	*n*	%
0–15	40,607,163	47.3	12,421,125	14.5	38,992,816	45.4
15–30	33,174,707	38.7	24,426,911	28.5	35,835,493	41.8
*Subtotal 0–30*	*73,781,870*	*86.0*	*36,848,036*	*43.0*	*74,828,309*	*87.2*
30–45	9,675,700	11.2	23,647,448	27.6	9,023,586	10.5
45–60	1,879,432	2.2	14,447,513	16.8	1,709,958	2.0
*Subtotal 30–60*	*11,555,132*	*13.4*	*38,094,961*	*44.4*	*10,733,544*	*12.5*
*Subtotal ≥ 60*	*474.633*	*0.6*	*10,868,638*	*12.6*	*249.782*	*0.3*
***Total***	***85,811,635***	***100.00***	***85,811,635***	***100.00***	***85,811,635***	***100.00***

### Palliative care units

**Fig. 1:**
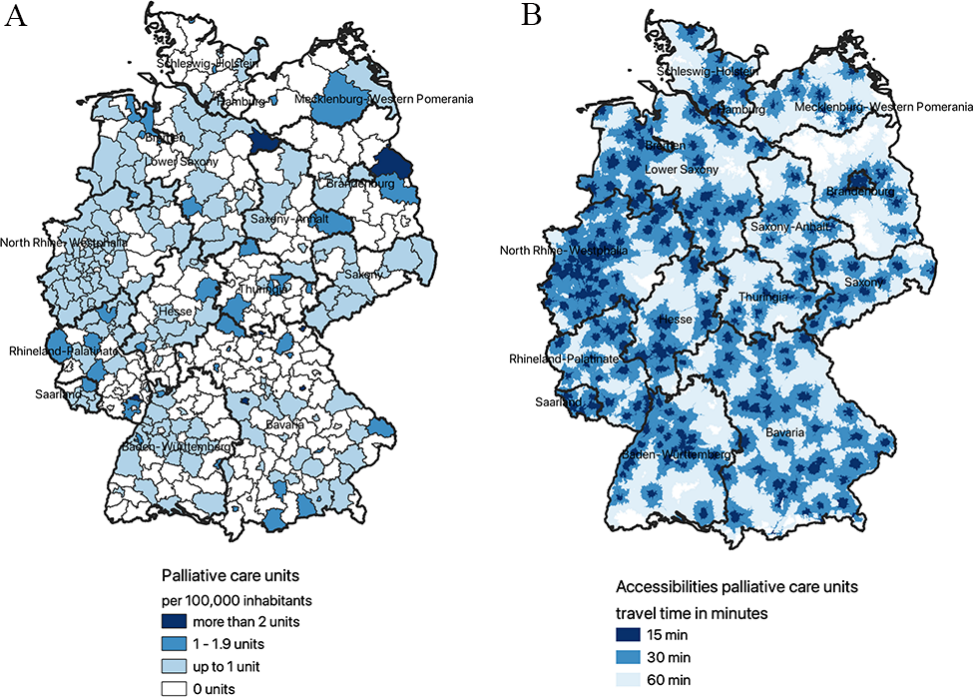
**A**. Distribution of PCU throughout Germany per 100,000 inhabitants on district level; **B**. Surface coverage of PCU throughout Germany

Of the 401 districts, 218 (54.4%) have at least one PCU with 142/218 (65.1%) being classified as urban. In 52 districts, more than one PCU exist. In total, 0.4 PCU are provided per 100,000 inhabitants (see Fig. 1.A). Figure 1.B displays the surface coverage resulting from the accessibility analysis based on the road network. In 94% of the area of Germany, a PCU can potentially be reached within a maximum of 60 minutes travel time by car and in 50% within a maximum of 30 minutes. With the centroid of all postal codes as an approximated starting point, residents need on average 22 minutes travel time to reach a PCU. This means that 99.4% of the population are living in areas where they can reach a PCU in less than 60 minutes and 86% of the people are living in areas where they must drive 30 minutes or less. Over 40 million people live in areas with less than 15 minutes driving time to the nearest PCU (see Table 1).

### Palliative care advisory teams in hospitals

**Fig. 2:**
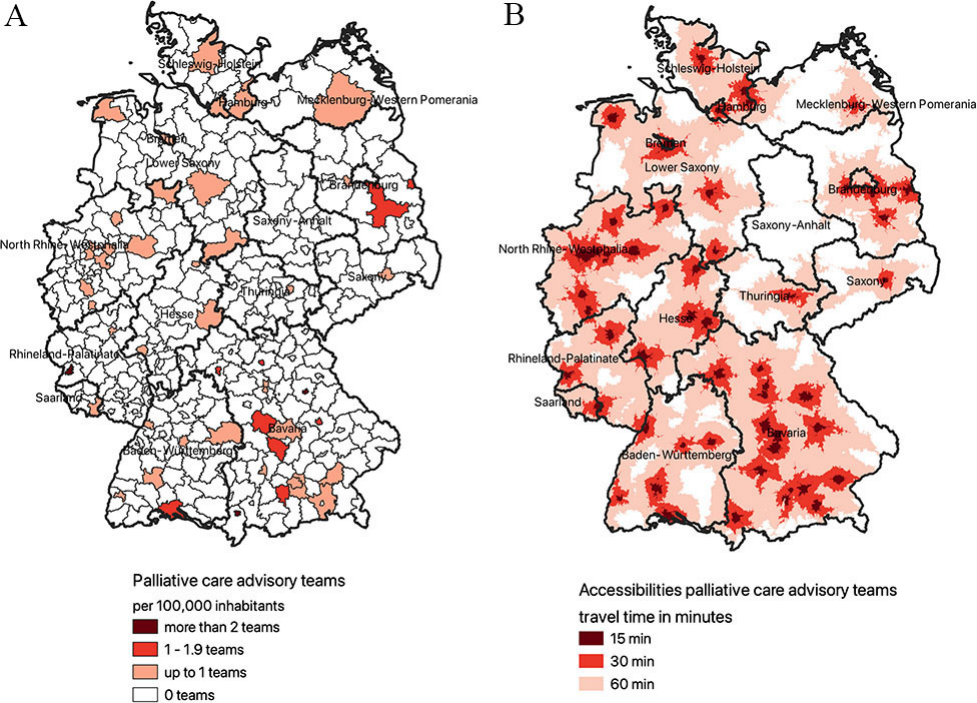
**A**. Distribution of PCA teams throughout Germany per 100,000 inhabitants on district level; **B**. Surface coverage of PCA teams throughout Germany

54/401 (13.5%) districts have hospitals providing a PCA team with 39/54 (72.2%) in urban areas (Fig. 2.A), and 0.1 teams per 100,000 inhabitants at all. A hospital with a PCA team can be reached in 60 minutes in 69% of the area of Germany and in 30 minutes in 17% (see Fig. 2.B). Residents would need to drive on average 40 minutes to hospitals with a PCA team. 12.6% of the population must travel more than one hour by car to reach the nearest hospital and 43.0% of all inhabitants can reach them within 30 minutes. Table 1 shows that one third of the population lives in areas with a travel time of 15–30 minutes to a PCA team.

### Specialist palliative home care teams

**Fig. 3:**
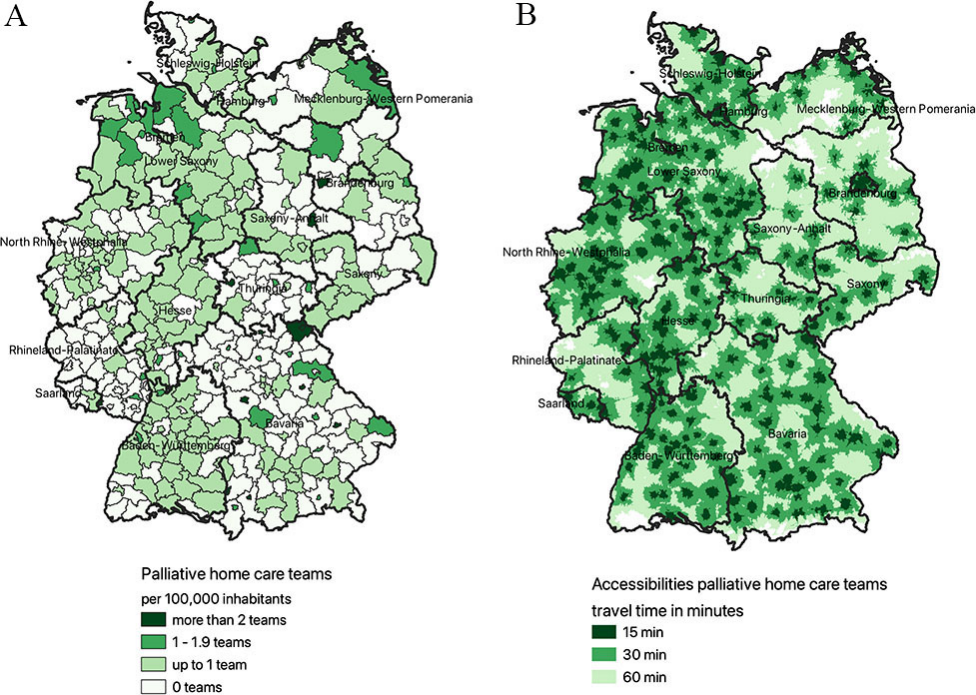
**A**. Distribution of SPHC teams throughout Germany per 100,000 inhabitants on district level; **B**. Surface coverage of SPHC teams throughout Germany

SPHC teams are established in 225/401 (56.1%) districts, with 127/225 (56.4%) defined as urban (Fig. 3.A). In total, there are 0.3 teams per 100.000 inhabitants. Accessibility analysis shows, that almost in the entire area (96%), a SPHC team can potentially reach patients within 60 minutes and in half of the area (54%) within 30 minutes (Fig. 3.B). SPHC teams need on average 20 minutes to reach a resident. 99.7% of the population are living in areas where they can be reached in less than 60 minutes and 87.2% in 30 minutes or less. Almost half of the population lives in the service area of a SPHC team with less than 15 minutes driving time.

## Discussion

We present one of the first national analysis of the distribution of SPC services in Germany, including population density and demonstrating a rather heterogeneous situation. The number of services per district varies by setting and district size, ranging from districts with no facility up to eleven services. Although there is uneven coverage of specialist services, most of the population lives within 0 to 30 minutes’ drive of a SPC service. The results demonstrate that the existing PCU and SPHC teams cover more than half of the area of Germany with an accessibility of 30 minutes and almost the whole country within 60 minutes. However, hospitals providing PCA teams can be reached within 30 minutes only in 17% of the total area of Germany, but with nearly half of the population living there. Furthermore, a quarter of all districts do not provide a specialist palliative care service at all. 96% of the population who do not have close access to hospitals with advisory teams can potentially access hospitals with PCUs. The 408,343 people who do not have access to an inpatient setting within an hour drive, can be reached by SPHC teams. Compared to 2010, the deficits in nationwide coverage identified at that time [16] have been greatly improved and the expansion of SPC services is still ongoing.

Palliative care and its continuity in the progression of the patient’s illness in various health care facilities is an integral part of all health care system structures [9]. Due to the setting-specific differences in patient groups and their needs, the different forms of SPC delivery cannot replace each other but should rather complement each other in the care of patients and should be offered close to residents’ homes. It should be noted that the quality of care in a region and its provision depends not only on the quality of individual services, but also on coordination and collaboration between specialist services and primary care providers. Organizing services into a coordinated regional network simplifies access to palliative care, helping to provide comprehensive care for patients and improve quality of life [29]. Early access to palliative care can not only improve quality of life [30] but is also cost-effective [31, 32]. It requires a close integration of all actors involved in the care of the individual patients. At the local and regional level, this is facilitated by hospice and palliative care networks [12].

Since the very beginning, the development and expansion of palliative care in Germany has come a long way over the past 20 years [12]. However, comparing services per resident, Germany is only middle ranked with 0.8 facilities (1.1 including inpatient hospices) per 100,000 residents [33]. According to a recent study, the need for palliative care in Germany is 5.9 facilities per 100,000 residents indicating a clear gap between the estimated need and the actual available services [34]. Comparing the potential accessibility e.g. to Ireland which has a better service per resident ratio (1.2 services per 100,00 residents), the surface coverage as well as the proportion of people living in an area of 30 minutes or less to drive is bigger [25]. In Spain, where the service per resident ratio is only 0.4 services per 100,000 residents, 79% of people living within a 30-minutes-drive, with a 30-minutes surface coverage of only 20%. In Switzerland, with a service per resident ratio of 1.1 services per 100,000 residents, 95% of the residents living within a 30-minutes-drive while only 40% of the surface has 30-minutes coverage to the nearest SPC service. This means, that the total amount of services is not the only determining factor for spatial distribution, but rather the location of the service in relation to the population distribution [25].

### Palliative care units

As our study shows, the distribution of PCU and their accessibilities are well developed. Over three quarter of the total population can potentially reach a PCU within 30 minutes. As already mentioned, a PCU is often used in acute situations and aims at crisis intervention and medical stabilization including the psychological and social support for patients and caregivers in a way that allows for discharge or transfer to another care setting [4] implying the need for short distances. The required number of palliative care beds in a particular region is generally dependent on regional demographics and socioeconomic trends, as well as the availability of other SPC services [9]. Consequently, the need for palliative care beds also depends on the availability and accessibility of services, such as those that provide outpatient and home care [8]. Although most residents who do not have access to a PCU within 30 minutes could potentially be cared for by a SPHC team, it is questionable whether, considering the primary purpose of PCU, all patient needs can be met.

### Specialist palliative home care

For home care service requirements, it is assumed that there should be one SPHC team for 100,000 residents,[8] but in fact there are only 0.3 teams available. There is evidence that most patients, in non-acute situations, prefer to be cared for in their own homes, if possible until they die. In contrast, most patients die in a hospital or nursing home [9]. There are specific regional factors like service and funding conditions for different palliative care services which result in regionally different service and team structures [35] and may explain different distribution patterns. In addition, there is an important role of general practitioners in rural areas, in providing SPHC services to patients. In rural areas in particular, the networking of existing resources appears to be crucial for the quality of patient care [14].

### Palliative care advisory teams

Complementary to PCU, the goal of PCA teams in hospitals is to improve symptom management, psychosocial support, and discharge management from acute units and to facilitate the transfer from inpatient to community care or to a PCU, depending on patients’ needs. Early integration of PCA teams in hospitals can facilitate the organization of patients’ care and relieve primary care providers [12]. PCA teams should be affiliated to PCU and should be available in all hospitals with more than 250 beds [9]. However, this support and care model is still very underrepresented in the majority of European countries, including Germany [9]. While 78% of the population can reach the nearest hospital (hospitals with at least 250 beds) within 15 minutes, only 15% of the population have access to the next PCA team within this time [36]. With regard to demographic changes and the increasing need for palliative care, there is insufficient coverage of PCA teams in hospitals across the whole country. Furthermore, the distribution of already established teams is very heterogeneous and there is no service provision in many areas and districts in Germany.

While we present new results on the accessibility of SPC in Germany, some limitations have to be considered. Besides the better understanding of the distribution of SPC services in Germany and their potential accessibilities, the geographic accessibility is only one part of the framework of access [37]. The analysis could not take into account the teams’ capacities and can therefore not allow conclusions regarding actual availability of the individual units and teams to patients. In addition, it remains an open question whether a maximum travel time of 60 minutes is appropriate as a threshold for accessibility to palliative care. An hour’s drive may be too long for relatives when someone is in the dying phase or for SPHC teams to take care for patients in acute situations.

### Strengths and weaknesses

This study has some strengths and weaknesses. First, the address data we used did not contain all SPC services in Germany. The Directory for Hospice and Palliative Care is the main resource for information on all SPC services in Germany and relies on self-reporting of services. However, recent estimations indicate that about 90% of the existing services are registered in the Directory [11] besides the data on PCA teams where the overall number is unknown. Second, we used travel times by car to approximate geographic accessibility. The alternative would be to consider travel distance, as used in Hesse et al. (2016) [38]. However, travel time is used more frequently in the literature and these two are highly correlated, [39] so this should not have a major impact on the results. Only allowing car travel time can, however, influence the results: If patients do not have access to a car or a potential caregiver driving them and rely on public transport, this would influence the results considerably. Third, no date and time were considered in the network analysis. The different possible travel times depending on the time of day or weather are acceptable limitations. The major strength is that we used official base maps with postal codes as smallest possible unit, which indicates the best possible population estimation included in the analysis.

## Conclusion

While PCU and SPHC teams are potentially accessible within a 30–60 minutes’ drive in many regions, PCA teams are not yet available in all relevant areas and hospitals. The collaboration and cooperation of the individual settings as a network is essential to provide the best quality of palliative care for patients with a life-limiting illness. Further implementation of PCA teams in hospitals is crucial to achieve these goals and enable early integration into specialist palliative care, as these teams are often the first entry point. For future studies and for the further spatial development of palliative care in Germany, it is necessary to conduct demand analysis for establishing new facilities and to link the indeed capacities and availabilities of the services with their potential accessibility.

## Electronic supplementary material

Below is the link to the electronic supplementary material.

**Additional file 1**. Characteristics of districts with and without facilities of Specialist Palliative Care

## Data Availability

Data on geographic information are freely available from the Service Center of the Federal Agency for Cartography and Geodesy at: https://gdz.bkg.bund.de/index.php/default/digitale-geodaten/. verwaltungsgebiete.html?___store = default. Data of the current spatial monitoring are freely available from the Federal Institute for Building, Urban Affairs and Spatial Research at: https://www.inkar.de/.
