# Supplementary material for: Accessibility to specialist palliative care services in Germany: a geographical network analysis
Source: BMC Health Serv Res. 2023 Jul 24;23:786. doi: 10.1186/s12913-023-09751-7 (PMC10364400; doi:10.1186/s12913-023-09751-7)
Supplement: Supplementary file 1 — Additional file 1. Characteristics of districts with and without facilities of Specialist Palliative Care [file 12913_2023_9751_MOESM1_ESM.docx]

Additional file 1. Characteristics of districts with and without facilities of Specialist Palliative Care

| **Districts** | **Settlement structures** | **PCU*** | | **PCA*** | | **SPHC*** | | **Total** | |
| --- | --- | --- | --- | --- | --- | --- | --- | --- | --- |
|  |  | n | % | n | % | n | % | n | % |
| *with facilities* |  | *218* | *54.3* | *54* | *13.5* | *225* | *56.1* | *291* | *72.6* |
|  | urban | 142 | 65.1 | 39 | 72.2 | 127 | 56.4 | 167 | 56.3 |
|  | rural | 76 | 34.9 | 15 | 27.8 | 98 | 43.6 | 124 | 42.6 |
| *without facilities* |  | *183* | *45.7* | *347* | *86.5* | *176* | *43.9* | *110* | *27.4* |
|  | urban | 56 | 30.6 | 159 | 45.8 | 71 | 40.3 | 31 | 28.2 |
|  | rural | 127 | 69.4 | 188 | 54.2 | 105 | 59.7 | 79 | 71.8 |

*PCU: Palliative Care Units; PCA: Palliative Care Advisory teams; SPHC: Specialist Palliative Home Care
